# An Efficient and Economical Protocol for Isolating, Purifying and PEG-Mediated Transient Gene Expression of Chinese Kale Hypocotyl Protoplasts

**DOI:** 10.3390/plants8100385

**Published:** 2019-09-28

**Authors:** Bo Sun, Qiao Yuan, Hao Zheng, Sha Liang, Min Jiang, Mei-Mei Wang, Qing Chen, Meng-Yao Li, Yong Zhang, Ya Luo, Rong-Gao Gong, Fen Zhang, Hao-Ru Tang

**Affiliations:** 1 College of Horticulture, Sichuan Agricultural University, Chengdu 611130, China; 14099@sicau.edu.cn (B.S.); 2018305010@stu.sicau.edu.cn (Q.Y.); 201607807@stu.sicau.edu.cn (H.Z.); 201607832@stu.sicau.edu.cn (S.L.); limy@sicau.edu.cn (M.-Y.L.); zhyong@sicau.edu.cn (Y.Z.);; 2 Institute of Pomology and Olericulture, Sichuan Agricultural University, Chengdu 611130, China

**Keywords:** Chinese kale, hypocotyl, transient expression, subcellular localization, *BaMYB75*

## Abstract

In this study, we report the isolation and purification of protoplasts from Chinese kale (*Brassica oleracea* var. *alboglabra*) hypocotyls, and their transient gene expression transformation and subcellular localization of *BaMYB75* (Bol042409). The upshot is that the vintage protocol included 5-d hypocotyls that were enzymatically hydrolyzed for 8 h in enzyme solution (3.0% cellulase, 0.5% pectolase, and 0.5 M mannitol), and the protoplasts were purified by precipitation. The total yield of protoplasts was 8 × 10^5^ protoplast g^−1^ fresh weight, and the protoplasts’ viability was 90%. The maximum transformation efficiency obtained by using green fluorescent protein (GFP) as a detection gene was approximately 45% when the polyethylene glycol (PEG)4000 concentration was 40% and transformation time was 20 min. In addition, *BaMYB75* was ultimately localized in the nucleus of Chinese kale hypocotyl protoplasts, verifying the validity and reliability of this transient transformation system. An effective and economical hypocotyl protoplast isolation, purification, and transformation system was established for Chinese kale in this study. This effectively avoided interference of chloroplast autofluorescence compared to using mesophyll cells, laying the foundation for future research in the molecular biology of *Brassica* vegetables.

## 1. Introduction

The plant protoplast refers to the entire cell without the cell wall, which is capable of absorbing foreign substances such as DNA plasmids and viruses [1,2,3,4]. Mechanical and enzymatic methods have been used to separate protoplasts. The process of mechanical protoplast separation is very complicated and the yield is low, while the enzymatic method easily separates a large number of protoplasts in a short time. Therefore, protoplasts are commonly separated by enzymatic hydrolysis. However, the optimal combination of enzyme solution and digestion time differs between species [5,6], making it necessary to develop an effective and suitable separation scheme for each species. 

Compared with stable gene expression in transgenic plants, transient gene expression is faster and more convenient [7,8]—especially using CRISPR/Cas9 technology—and the advantage of transient gene expression in gene target site screening is clear [9,10]. The currently used transient transformation method is polyethylene glycol (PEG)-mediated transient expression of protoplasts [11]. Effective mesophyll protoplast isolation, purification, and transient transformation systems have been established in many plant species, such as wheat [12], *Arabidopsis thaliana* [11], and Chinese kale [13]. Studies on plant hypocotyl protoplasts have focused on isolation, purification, and regeneration [14,15,16,17]. There are seldom reports on the transient transformation of hypocotyl protoplasts, particularly in *Brassica* vegetables.

Chinese kale is a *Brassica* species in the Brassicaceae family. Chinese kale is promoted in many parts of China because of it is rich in nutrients such as vitamin C, glucosinolates, and carotenoids. A protoplast isolation, purification, and transient transformation system for Chinese kale mesophyll was established in our previous study [13], but there were some limitations in the application process. For example, mesophyll cells are generally small, which is not conducive to observation. When observed under a fluorescence microscope, the autofluorescence of chloroplasts in mesophyll cells interferes with the visual field. However, this problem can be solved by using Chinese kale hypocotyl protoplasts. Hypocotyl cells are larger than mesophyll cells and there is no interference from chloroplasts, so the results of localization can be clearly seen using a low-cost ordinary fluorescence microscope rather than expensive confocal microscopy. It is particularly important to establish a highly efficient protoplast isolation, purification, and transient transformation system for the Chinese kale hypocotyl.

In this study, Chinese kale hypocotyls were used as the test material to study the effects of combining enzyme solution, digestion time, mannitol concentration, hypocotyl seedling age, and purification method on hypocotyl protoplast isolation and purification in Chinese kale. The effects of PEG concentration and transformation time on transient transformation were investigated using green fluorescent protein (GFP) as the reporter gene. The viability and effectiveness of the system were demonstrated in Chinese kale via subcellular localization of *BaMYB75,* a member of the *MYB* transcription factor family.

## 2. Results

### 2.1. Effects of Enzyme Composition on Isolating Protoplast from Chinese Kale Hypocotyls

**Figure 1:**
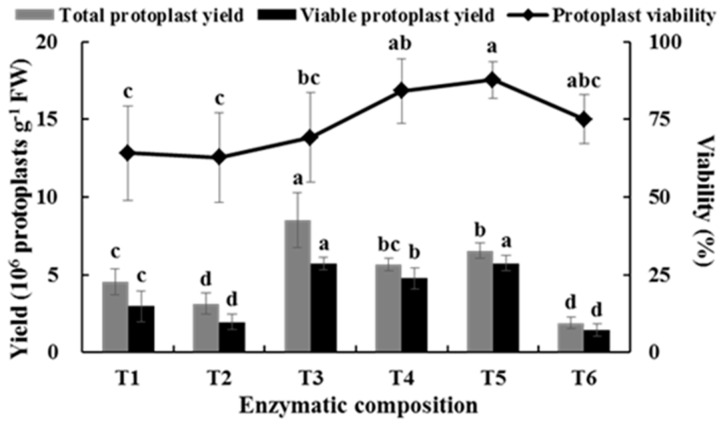
Effects of enzyme composition on the total yield and viability of Chinese kale hypocotyl protoplasts. Different letters represent a significant difference at *p* ≤ 0.05, and bars represent standard errors (SEs) (same as below). T1: 1.0% cellulose + 0.3% pectolase; T2: 1.0% cellulose + 0.5% pectolase; T3: 1.0% cellulose + 0.7% pectolase; T4: 3.0% cellulose + 0.3% pectolase; T5: 3% cellulose + 0.5% pectolase; T6: 3% cellulose + 0.7% pectolase.

Different enzyme compositions affected the yield and viability of the hypocotyl protoplasts in Chinese kale (Figure 1). When the cellulase concentration was 1.0%, the yield of protoplasts in 0.7% pectolase was 8.52 × 10^6^ protoplasts g^−1^ FW, which was significantly higher than those of 0.3% and 0.5% pectolase. However, no significant difference was observed in the viability of protoplasts among the above treatments. When the concentration of cellulase was 3.0%, the protoplast yields under 0.3% and 0.5% pectolase were significantly higher than that of 0.7%. Although no significant difference was detected in the total yield and viability of protoplasts between 0.3% and 0.5%, the viable protoplast yield was higher when 0.5% pectolase was used. The viable protoplast yields for the combinations of 1.0% cellulase and 0.7% pectolase and 3.0% cellulase and 0.5% pectolase were not significantly different, but the viability of the latter was significantly higher than that of the former. Therefore, the combination of 3.0% cellulase and 0.5% pectolase was determined to be the optimal combination of enzymes to isolate protoplasts from Chinese kale hypocotyls.

### 2.2. Effects of Digestion Time on Isolating Protoplasts from Chinese Kale Hypocotyls

**Figure 2:**
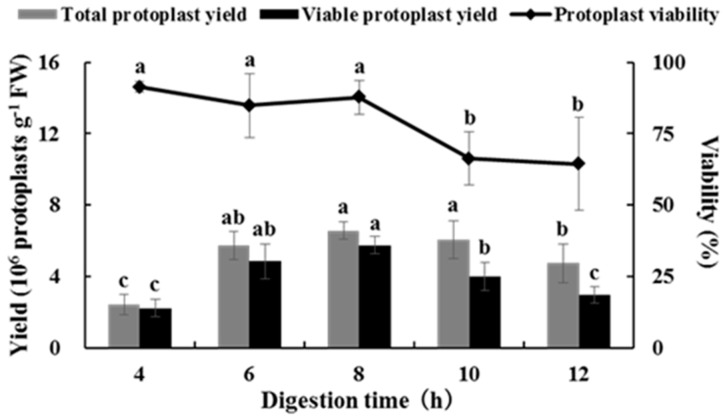
Effects of digestion time on total yield and viability of Chinese kale hypocotyl protoplasts.

Different digestion times affected the yield and viability of the hypocotyl protoplasts from Chinese kale. The protoplast yield first increased and then decreased with an increase in digestion time. The yield reached a maximum (6.55 × 10^6^ protoplasts g^−1^ FW) after digestion for 8 h, and the protoplast viability reached 87% (Figure 2). The results showed that high yield and high viability of protoplasts could be obtained by using the combination of 3.0% cellulase and 0.5% pectolase for 8 h.

### 2.3. Effects of Mannitol Concentration on Isolating Protoplasts from Chinese Kale Hypocotyls

**Figure 3:**
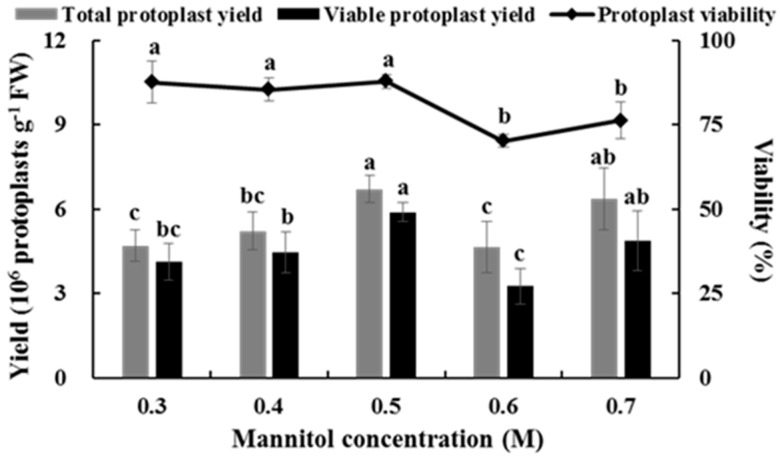
Effects of mannitol concentration on the yield and viability of Chinese kale hypocotyl protoplasts.

The concentration of mannitol had a strong effect on the yield and viability of protoplasts, and the yield of protoplasts increased gradually along with increasing mannitol concentration. When the concentration of mannitol was increased to 0.5 M, protoplast yield reached its maximum (6.71 × 10^6^ protoplasts g^−1^ FW). However, protoplast yield decreased significantly when mannitol concentration was increased to 0.6 M. Although protoplast yield increased in 0.7 M mannitol, protoplast viability was significantly lower than that at 0.5 M mannitol (Figure 3). Therefore, the suitable mannitol concentration for isolating protoplasts from Chinese kale hypocotyls was 0.5 M.

### 2.4. Effects of Seedling Age on Isolating Protoplasts from Chinese Kale Hypocotyls 

**Figure 4:**
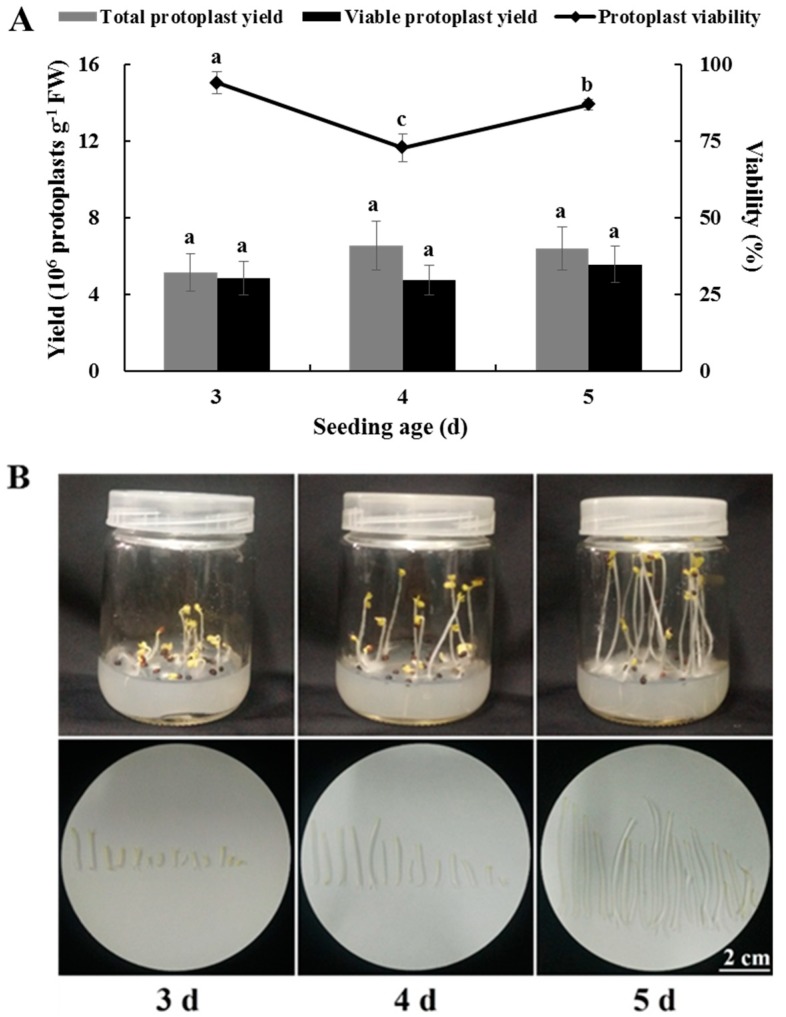

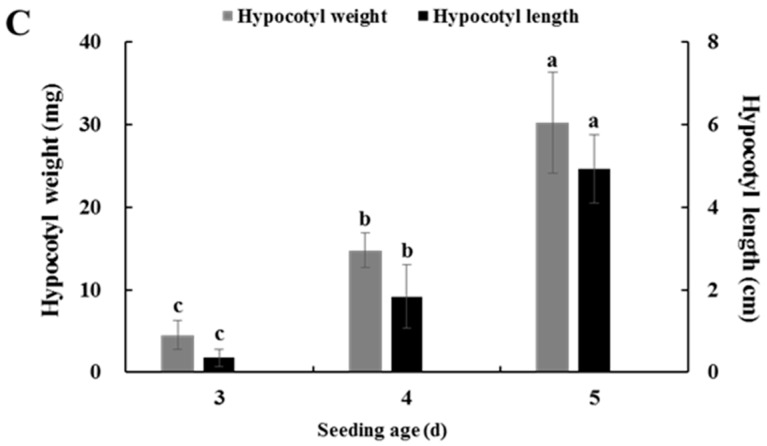
Effects of seedling age on yield, viability, hypocotyl growth status and hypocotyl length, and weight of Chinese kale hypocotyl protoplasts. (**A**) Yield and viability; (**B**) Hypocotyl growth status; (**C**) Hypocotyl length and weight. Scale bars in (B) = 2 cm.

No significant differences were observed in total or viable yields of hypocotyl protoplasts after 3, 4, and 5 d. Although viability of the hypocotyl protoplasts at 3 d was significantly higher than that at 5 d (Figure 4A), hypocotyl weight and hypocotyl length after 5 d were 6.7-fold and 14.2-fold those after 3 d, respectively (Figure 4B,C). Considering the cost and utilization of material, it was finally determined that 5 d was the optimum seedling age to isolate protoplasts from Chinese kale hypocotyls.

### 2.5. Effects of Purification Method on Isolating Protoplasts from Chinese Kale Hypocotyls 

**Figure 5:**
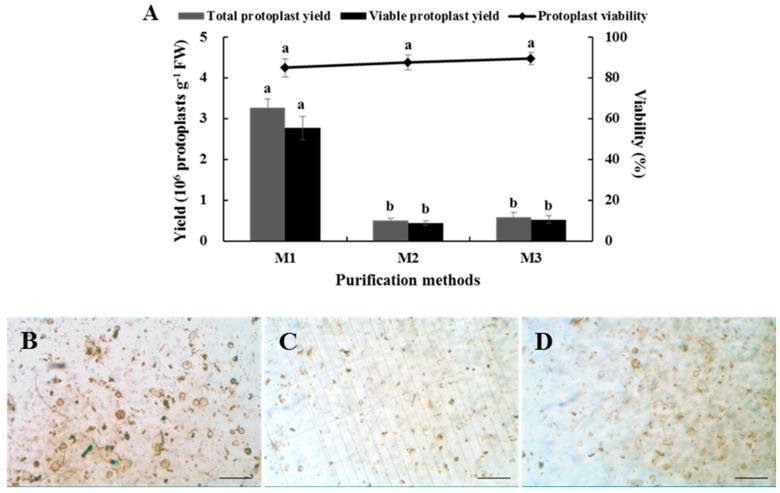
Effects of purification method on yield, viability, and purity of Chinese kale hypocotyl protoplasts. (**A**) Resulting yield and viability. M1: before purification; M2: floating method; M3: precipitation method. Light microscopy images (**B**) Before purification; (**C**) After purification by floating method; (**D**) After purification by precipitation method. Scale bars in (B–D) = 200 μm.

A suitable purification method is capable of obtaining high-quality protoplasts. The results showed that a large number of protoplasts were lost by both methods, and there was no significant difference in the yield or viability of the protoplasts obtained between them (Figure 5A). However, the precipitation method produced less damage to large cells (Figure 5B–D), and was more convenient to operate, so the precipitation method was determined to be more suitable for purifying Chinese kale hypocotyl protoplasts.

**Figure 6:**
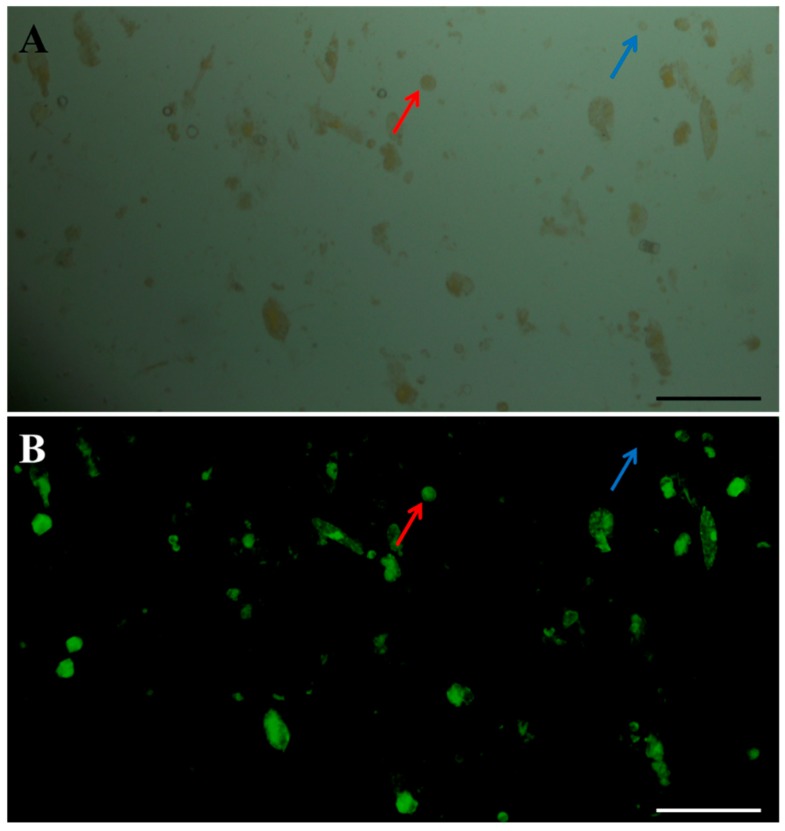
Viability of protoplasts isolated from Chinese kale hypocotyls: (**A**) Fluorescein diacetate (FDA)-dyed protoplasts under bright light and (**B**) FDA-dyed protoplasts under ultraviolet light. Scale bars = 100 µm. Red arrows represent viable protoplasts and blue arrows represent non-viable protoplasts.

In short, an optimized isolation and purification protocol for Chinese kale hypocotyl protoplasts was obtained as follows. Five-day hypocotyls were hydrolyzed in an enzyme solution containing 3.0% cellulase and 0.5% pectolase and a mannitol concentration of 0.5 M for 8 h. The total yield of protoplasts purified by the precipitation method was 8 × 10^5^ protoplasts g^−1^ FW, and protoplast viability was determined to be 90% by the fluorescein diacetate (FDA) staining analysis (Figure 6).

### 2.6. Transformation Efficiency of Chinese Kale Hypocotyl Protoplasts

**Figure 7:**
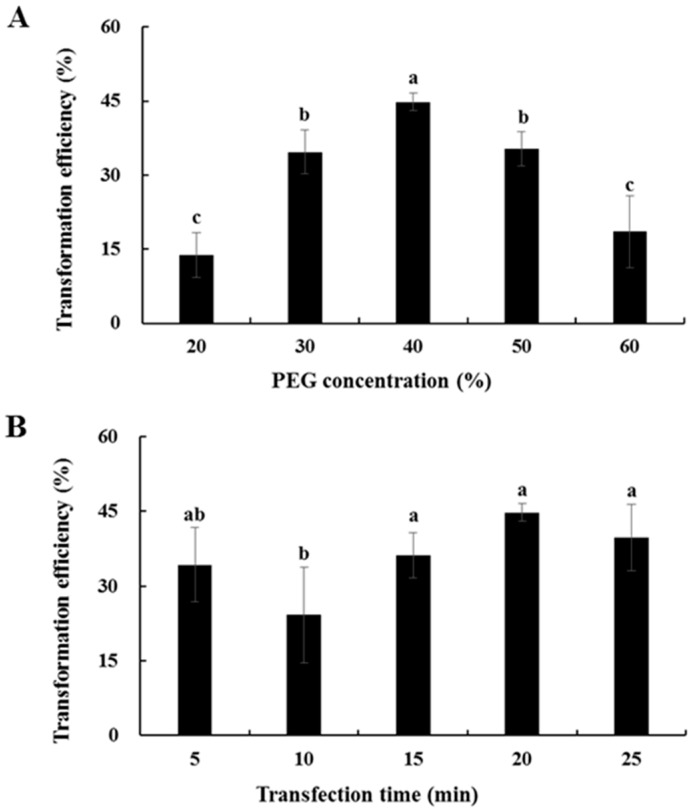
Effects of PEG4000 concentration and transformation time on protoplast transformation efficiency: (**A**) PEG4000 concentration; (**B**) Transformation time.

The effects of PEG4000 concentration and transformation time on the transformation efficiency of Chinese kale hypocotyl protoplasts were evaluated using the GFP vector. Transformation efficiency first increased and then decreased as the concentration of PEG4000 was increased (Figure 7A). When the concentration of PEG4000 was 40%, transformation efficiency attained the maximum of approximately 45%, but a higher concentration of PEG4000 (50% or 60%) resulted in a gradual decline in transformation efficiency. As shown in Figure 7B, the highest transformation efficiency was found after 20 min of transformation, and the level was approximately 45%. Above all, the optimal transformation scheme for Chinese kale hypocotyl protoplasts was performed in 40% PEG4000 for 20 min in the dark.

### 2.7. Subcellular Localization of the GFP-Fused BaMYB75 Protein in Chinese Kale Hypocotyl Protoplasts

**Figure 8:**
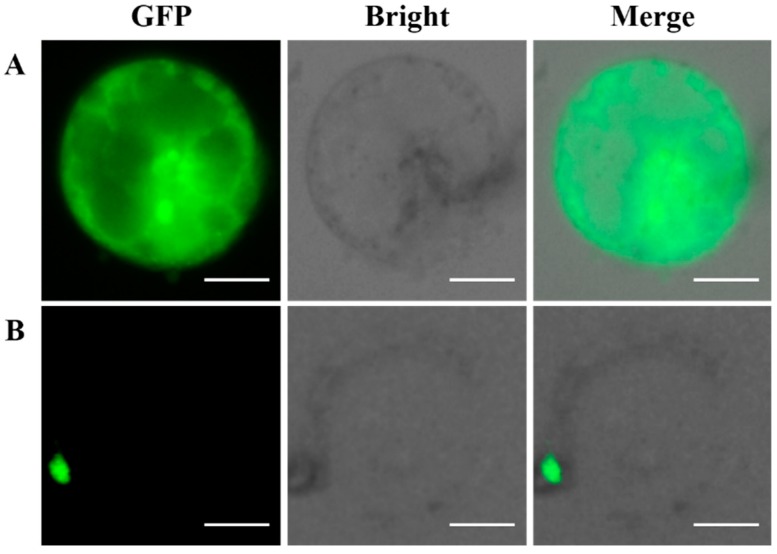
Subcellular localization analysis of the (**A**) green fluorescent protein (GFP) only and (**B**) MYB10–GFP vectors in Chinese kale hypocotyl protoplasts under a fluorescence microscope. Scale bars = 20 µm.

The subcellular localization of BaMYB75 fused to the GFP vector in Chinese kale hypocotyl protoplasts was investigated to verify our transient expression system. A clear GFP signal was specifically detected in the nucleus, when the GFP–BaMYB75 (C-terminus) fusion protein was co-expressed in the Chinese kale hypocotyl protoplasts (Figure 8B), which was consistent with the predicted results. As a comparison, the expression of the GFP reporter gene was clearly detected in all organelles (Figure 8A). These findings confirm that Chinese kale hypocotyl protoplasts are suitable for subcellular localization experiments.

## 3. Discussion

In this study, we developed an isolation and purification protocol as well as an efficient PEG-mediated transient expression system for Chinese kale hypocotyl protoplasts, and the system was successfully applied to localize the *BaMYB75* gene. The *MYB* gene family is a family of transcription factors usually located in the nucleus that regulates plant development and metabolism [18,19], such as the sweet cherry *PacMYBA* gene [20]. In the present study, *BaMYB75*—which is homologous to *PacMYBA* in Chinese kale—was cloned. The results showed that the *BaMYB75* gene was specifically detected in the nucleus of hypocotyl protoplasts. The localization results were clear at first glance due to the absence of interference from chloroplast autofluorescence.

Different species, even different organs in the same plant, have different enzyme compositions that affect the preparation of protoplasts [21]. The suitable enzyme combination for Chinese kale hypocotyl protoplasts was 3.0% cellulase and 0.5% pectolase, while the enzyme combination suitable for mesophyll protoplasts was 2.0% cellulase and 0.1% pectolase [13]. Thus, the suitable cellulase and pectolase concentrations for hypocotyl protoplasts were higher than those for mesophyll protoplasts. It has been reported that the cellulose and pectin contents in the cell wall material of tobacco stems are much higher than those in leaves [22]. We speculate that the cellulose and pectin contents in the Chinese kale hypocotyl cell wall may be higher than those in mesophyll. Thus, the concentrations of cellulase and pectolase in the enzyme solution for the hypocotyl cells were higher than those for mesophyll cells. On the other hand, in other species such as chestnut [23] and cucumber [24], the suitable enzyme combination for hypocotyls is significantly different than that of Chinese kale, which may be due to a difference in plant species [25].

Different mannitol concentrations affected the yield and viability of protoplasts, while different organs of the same plant have different adaptabilities to mannitol [26,27,28]. In this study, the appropriate mannitol concentration for hypocotyls was lower than that for mesophyll [13]. There are seldom chloroplasts in hypocotyl cells grown in the dark—rather, most have only a vacuole and a few organelles. The internal structure of hypocotyl cells is loose [29] and the osmotic pressure is rather low. However, in mesophyll cells the vacuole is small or even absent. Most cells contain more chloroplasts [30] with smaller and denser intracellular spaces than those of hypocotyl cells, meaning they can withstand greater osmotic pressure. Therefore, it is speculated that the osmotic pressures tolerated by the two are also different due to differences in spatial composition between hypocotyl cells and mesophyll cells, resulting in a difference in the suitable mannitol concentration.

The seedling age of the hypocotyl has an effect on the yield and viability of protoplasts. For example, the yield and viability of kenaf hypocotyl protoplasts under 3 d of darkness were significantly higher than those at 5, 7, 9 and 11 d [31]. A similar result was reported in violet, in which the yield and viability of 14-d violet hypocotyl protoplasts were significantly higher than those of other days [32]. However, the results of the present study produced no differences in the yield of Chinese kale hypocotyl protoplasts among 3, 4 and 5 d. This could be due to the shorter time interval for culturing hypocotyls and the distinct species.

At present, the protoplast purification methods are usually precipitation and floatation. The floating method has been used in cabbage [33] and grape [34], while the precipitation method has been adopted in Tartary buckwheat [35]. However, no study has compared the two methods. In this study, the two methods were compared and evaluated, and the results indicated that the precipitation method was more suitable for Chinese kale hypocotyl protoplasts because it caused less damage to the Chinese kale’s large cells. However, many Chinese kale hypocotyl protoplasts were lost during both methods. Impurities such as cell debris also occurred after purification, which might have interfered with observations during FDA staining and subcellular localization test. Therefore, optimizing the protoplast purification method and reducing the effect of hypocotyl protoplast impurities are directions for future studies.

In this study, the total yield of Chinese kale hypocotyl protoplasts purified was 8 × 10^5^ protoplasts g^−1^ FW, and protoplast viability was about 90%. These results are similar to those of the hypocotyls of chestnut [23], carrot [17], kenaf [31], and *Matthiola incana* [32]. Moreover, there are significant differences of transformation efficiency among different plant species. The range of transformation efficiency of some fruits and vegetables, such as sweet cherry [20], Chinese kale [13], cucumber [24], and *Phaseolus vulgaris* [36], is from 30% to 85%. In our study, the transformation efficiency of hypocotyl protoplasts obtained by the optimized separation, purification, and transient transformation method was 45%, which is within this effective range. Considering the yield, viability, and transformation efficiency, this protocol on Chinese kale hypocotyl is efficient.

Observations of protoplasts expressing the GFP fusion protein can be carried out under ordinary fluorescence microscopy or fluorescence confocal microscopy. Confocal microscopy is one of the most advanced molecular cell biology analytical instruments. The imaging effect is three-dimensional and clear, but running the test is very costly. Fluorescence microscopes are more widely used in many laboratories, the cost of which is less than 20% than that of confocal microscopy. However, when cells containing chloroplasts are observed under a fluorescence microscope, the autofluorescence of the chloroplasts causes intense interference which is not conducive to the observations. In the present study, hypocotyl protoplasts with few chloroplasts were used for the localization test and were effectively observed by ordinary fluorescence microscopy without any interference from chloroplast autofluorescence. This indicates that this is an economical protocol.

## 4. Materials and Methods 

### 4.1. Plant Materials

The Chinese kale “Cutiaoyusun” cultivar was used in this study. Mature Chinese kale seeds were immersed in 75% (*v/v*) alcohol for 30 s after a warm water bath and then immersed in 0.1% (*w/v)* mercury chloride for 6 min, rinsed with sterile distilled water, and dried with sterilized filter paper. The sterilized seeds were germinated on 1/2 Murashige and Skoog medium (2.0% (*w/v*) sucrose, 0.8% (*w/v*) agar, pH 5.8) in 240-mL tissue culture flasks, with 30 seeds per flask. The seedlings were maintained under dark conditions at 25 °C to obtain the hypocotyls to isolate the protoplasts.

### 4.2. Protoplast Isolation and Purification

Hypocotyls of different seedling ages (3, 4 and 5 d) were used as the materials to optimize the hypocotyl seedling age. The hypocotyls were cut into thin slices (0.5–1.0 mm), pretreated in 10 mL of 0.5 M mannitol (Solarbio, Beijing, China) solution for 1 h, and then placed in an enzyme solution for hydrolysis. The enzyme solution contained mannitol and 5 mM 2-(N-morpholino) ethanesulfonic acid (MES) (Macklin, Shanghai, China) that was dissolved in cell wash medium salts. Afterwards, different concentrations of cellulase R-10 (Sangon, Shanghai, China) (1.0% and 3.0%) and pectolase Y-23 (Sangon, Shanghai, China) (0.3%, 0.5% and 0.7%) were added to optimize the concentration of mannitol. Different concentrations of mannitol (0.3, 0.4, 0.5, 0.6 and 0.7 M) were added into the enzyme solution. All enzyme solutions were adjusted to pH 5.8 and filter-sterilized through a 0.22-µm syringe filter. The pretreated hypocotyls were treated with 10 mL of the enzyme solution and incubated in the dark at 25 °C with gentle shaking (45 rpm). To optimize digestion time, different digestion times (4, 6, 8, 10 and 12 h) were tested. Then, the enzyme mixture was filtered through 300 mesh nylon. To optimize the purification method, the protoplasts were purified using precipitation [37] and floating methods [33], and the yield and viability of protoplasts under these two methods were compared.

Protoplast yield was determined using a double-chambered hemocytometer and an Olympus CX21 light microscope (Olympus Inc., Tokyo, Japan). The viability of the protoplasts was determined by staining with 0.01% (*w/v*) fluorescein diacetate (FDA) (Yuanye, Shanghai, China) [38]. Protoplast yield and viability were calculated as follows: protoplast yield = number of protoplasts in the enzyme solution/fresh weight of the plantlet leaves used in the enzyme solution; protoplast viability (%) = (number of the fluorescent protoplasts in view/number of the total protoplasts in view) × 100%.

### 4.3. Protoplast Transformation 

Isolated protoplasts were held on ice to precipitate for 30 min. The upper liquid was then discarded and the protoplasts were resuspended in MMG solution (0.4 M mannitol, 15 mM MgCl_2_ (Kelong, Chengdu, China), 4 mM MES, pH = 5.7) at room temperature. A weight of 10 µg pC2300-35S-GFP plasmid DNA was added to 100 µL of the prepared protoplasts and mixed gently. An equal volume of freshly prepared PEG4000 solution (PEG4000 (Biotopped, Beijing, China), 0.2 M mannitol, and 100 mM CaCl_2_ (Kelong, Chengdu, China)) was added immediately and mixed carefully. To optimize the PEG concentration, the transformation efficiency was examined at different concentrations of PEG4000 (20%, 30%, 40%, 50% and 60%, (*w/v*)). The mixture was incubated for 5, 10, 15, 20 and 25 min in the dark at room temperature to optimize the transformation duration. After the incubation, the transformation process was stopped by adding 550 μL of W5 solution. The mixture was centrifuged at 1000 r min^−1^ for 2 min and the protoplasts were gently resuspended in 1 mL WI solution (4 mM MES, 0.5 M mannitol, 20 mM KCl (Kelong, Chengdu, China), pH = 5.7). The transfected protoplasts were incubated at 25 °C in the dark for 24 h and centrifuged at 1000 r min^−1^ for 2 min, then resuspended with an appropriate amount of WI solution.

The protoplasts expressing the GFP-fusion protein were observed and images were captured using an Olympus BX-51 fluorescence microscope equipped with a DP70 camera (Olympus, Japan). The protoplasts were observed with a 40× oil objective for subcellular localization analysis. Transformation efficiency (%) = (fluorescent protoplast number in view/total protoplast number in view) × 100%.

### 4.4. Subcellular Localization

To evaluate usability of the system, the *BaMYB75* gene (GenBank Accession no: Bol042409) in Chinese kale was selected. pC2300-35S-BaMYB75-GFP was constructed for subcellular localization. The complete coding sequence (CDS) of *BaMYB75* was amplified using the primers BolMYB75-GFP-F: 5ʹ-cg**GGATCC**ATGGAGGATTCGTCCAAAGGGTT-3ʹ (The bold font is the *BamH* I restriction site, and the lowercase letters represent the restriction site to protect the base) and BolMYB75-GFP-R: 5ʹ-acgc**GTCGAC**ATCAAGTTCTACAGTCTCTCCATCCAACA-3ʹ (The bold font is the *Sal* I restriction site, and the lowercase letters represent the restriction site to protect the base), in which a *BamH* I site at the 5ʹ-end and a *Sal* I site at the 3ʹ-end of the gene were incorporated, respectively. After double digestion, the gene was inserted into the empty vector and GFP was fused in frame at the *BaMYB75* C-terminal. Finally, the recombinant plasmid pC2300-35S-BaMYB75-GFP was transformed into Chinese kale protoplasts according to the optimized protocol. Subcellular localization was forecasted by WoLF PSORT software (http://www.genscript.com/wolf-psort.html).

### 4.5. Data Analysis

All experiments were replicated three times. The statistical analysis was performed with SPSS Version 18.0 software (SPSS Inc., Chicago, IL, USA). Significant differences among treatments were determined at *p* ≤ 0.05 based on the least significant difference test. Data are presented as mean ± standard error from three independent experiments.

## 5. Conclusions

An efficient and economical isolation and purification system for Chinese kale hypocotyl protoplasts was established. The high yield and high viability of the protoplasts obtained using our protocol can be used for further studies of gene functions in Chinese kale. In addition, a transient transformation system for Chinese kale hypocotyl protoplasts was established in this study, which effectively eliminated the interference of chloroplast autofluorescence in the subcellular localization experiment. In short, this protocol can achieve ideal results and is also cost effective.
